# Evaluation of adverse effects of chemotherapy regimens of 5-fluoropyrimidines derivatives and their association with DPYD polymorphisms in colorectal cancer patients

**DOI:** 10.1186/s12885-020-06904-3

**Published:** 2020-06-16

**Authors:** Reza Negarandeh, Ebrahim Salehifar, Fatemeh Saghafi, Hossein Jalali, Ghasem Janbabaei, Mohammad Javad Abdhaghighi, Anahita Nosrati

**Affiliations:** 1 Department of Pharmaceutics, Facuity of Pharmacy, Mazandaran University of Medical Sciences, Sari, Iran; 2 Pharmaceutical Research Center, Hemoglobinopathy institute, Department of Clinical Pharmacy, Mazandaran University of Medical Sciences, Sari, Iran; 3 Department of Clinical Pharmacy, Faculty of Pharmacy and Pharmaceutical Sciences Research Center, Shahid Sadoughi University of Medical Sciences, Yazd, Iran; 4 Thalassemia Research Center, Mazandaran University of Medical Sciences, Sari, Iran; 5 Gastrointestinal Cancer Research Center, Faculty of Medicine, Mazandaran University of Medical Sciences, Sari, Iran; 6 Department of Pharmacognosy, Facuity of Pharmacy, Mazandaran University of Medical Sciences, Sari, Iran; 7 Department of Pathology, Imam Khomeini Hospital, Mazandaran University of Medical Sciences, Sari, Iran

**Keywords:** Colorectal cancer, 5-fluorouracil, Polymorphism, Dihydropyrimidine dehydrogenase, Side effects

## Abstract

**Background:**

5-Fluorouracil (5-FU) and capecitabine are fluoropyrimidine derivatives that mainly metabolized with dihydropyrimidine dehydrogenase enzyme (DPD). The genetic polymorphism in the genes encoding this enzyme may result in a decrease or loss of enzyme activity which may lead to the accumulation of medicines, their metabolites and potential toxicity.

**Method:**

This cross-sectional study was conducted on 88 participants with colorectal cancer (CRC). After DNA extraction, polymerase chain reaction-restriction fragment length polymorphism (PCR-RFLP) method was used to determine the DPD gene *(DPYD)* polymorphisms including IVS 14 + 1 G > A, 2846 A > T and 2194 G > A. Chemotherapy-induced side effects were evaluated according to the Common Terminology Criteria for Adverse Events (CTCAE Version 5.0).

**Result:**

Data were collected from 227 chemotherapy cycles of 88 patients with CRC. In a comparison of FOLFOX and FOLFIRI regimens, there was no significant difference in the occurrence of chemotherapy-induced diarrhea, nausea, vomiting and oral mucositis. However, the peripheral neuropathy was more frequent in patients who were treated with FOLFOX (*P* <  0.001) and hair loss was more common in patients who received FOLFIRI regimen (*P* = 0.048).

Incidence of the DPD IVS14 + 1 G > A polymorphism was observed in four patients (5.5%). There was no association between IVS14 + 1 G > A polymorphism and the occurrence of adverse reactions.

**Conclusion:**

FOLFOX and FOLFIRI were the most common regimens in CRC patients and their toxicity profile was different in some adverse reactions. Prevalence of IVS14 + 1G > A variant was relatively higher than other similar studies.

**Trial registration:**

Approval code; IR.MAZUMS.REC.95.2480.

## Background

Nearly 50,000 incident cancer cases are reported in Iran annually. It has been reported that gastrointestinal (GI) cancers have the highest incidence rate in Iran [1]. CRC is the third most frequent cancer in the worldwide and is ranked third in terms of mortality [2]. There are different types of cancer treatments, including surgery, targeted therapies, and chemotherapy. The choice of therapy depends upon several factors such as tumor stage, molecular tumor indices, and functional status of the patient [3]. Fluoropyrimidine derivatives including 5-FU and capecitabine (prodrug of 5-FU) inhibit the synthesis of thymidylate synthase (TS) leading to inhibition of synthesis of purine and pyrimidine bases [4–6]. 5-FU is a cytotoxic chemotherapy medicine that is widely used in the treatment of a variety of cancers, including colon, rectum, breast, stomach, and pancreas [7]. A substantial number of enzymes are involved in the metabolism of 5-FU, First, thymidine phosphorylase (TP) catalyzes the conversion of 5-FU to its active metabolite, 5-fluoro-2′-deoxyuridine. Subsequently, 5′-fluoro-2-deoxyuridine converts to its active metabolite 5-fluoro-2′-deoxyuridine 5′-monophosphate (FdUMP) through phosphorylation by the thymidine kinase. FdUMP forms a ternary complex with methylene tetrahydrofolate and TS which finally lead to the inhibition of DNA synthesis. By inhibiting the participation of uracil in RNA, the complex will also inhibit RNA synthesis [5–8]. DPD is an enzyme present in the liver and accountable for about 80–85% of 5-FU catabolism. DPD catabolizes 5-FU to 5,6-dihydro-5-fluorouracil (DHFU) [5–9]. DPD deficiency can be associated with an exacerbation of diarrhea, nausea, vomiting, mucositis, and neurotoxicity of fluoropyrimidine derivatives [10]. The DPD enzyme is encoded by the *DPYD* and several single nucleotide polymorphisms (SNP) including IVS14 + 1G > A, 464 T > A, 2194 G > A, 496 A > G, and 1627 A > G have been reported in the DPYD [11–13].

DPYD deficiency following absence or mutation of the allele reduces the 5-FU clearance and may increase the risk of developing severe toxicity. Mutation in some variants such as IVS14 + 1 G > A, 2846 A > T and 2194 G > A may lead to reduced DPD enzyme levels [14].

The most common type of genetic polymorphism reported in patients with CRC that leads to a decrease or elimination of the activity of the DPD enzyme is mutation from guanine to adenine in *DPYD* of intron 14 called rs3918290 or IVS14 + 1G > A or * 2 A [15]. In addition to IVS14 + 1G > A, other polymorphisms including 464 T > A and 2194 G > A have also been associated with 5-FU side effects such as bone marrow depression and digestive tract complications [16]. Different chemotherapy regimens have been used in the treatment of CRC including FOLFOX (5-FU; 400 mg/m^2^ IV bolus over 5 min and 2400 mg/m^2^ IV over 46 h; leucovorin 400 mg/m^2^ IV day 1 and oxaliplatin 85 mg/m^2^ IV day 1), FOLFIRI (5-FU 400 mg/m2 IV bolus over 5 min and 2400 mg/m2 IV over 46 h; leucovorin 400 mg/m^2^ IV and irinotecan 180 mg/m^2^ IV), and XELOX (capecitabine 1000 mg/m2 twice daily for 14 of every 21 days and oxaliplatin 130 mg/m^2^ on day 1) [17, 18].

The frequency of occurrence and severity of side effects of 5-FU and capecitabine may vary according to the type of chemotherapy regimen and interpersonal differences in the expression of genes encoding enzymes involved in the metabolism of drugs.

Further studies are required to have a better understanding of the association between *DPYD* polymorphism and the side effects of chemotherapy regimens of 5-FU derivatives [19]. This study aimed to evaluate the adverse effects of different chemotherapy regimens used in CRC patients and the relationship between genetic polymorphism of *DPYD* and the adverse effects of chemotherapy regimens in a sample of CRC patients in the north of Iran.

## Methods

From October of 2016 to June of 2017, this cross-sectional study was carried out in outpatient oncology clinic of Imam Khomeini Hospital, Sari, Mazandaran. Eighty-eight patients with colon or rectal cancer were randomly assigned to receive either 5-FU or capecitabine in common chemotherapy regimens in use for CRC, including FOLFOX and FOLFIRI. Some of the patients were unable to continue the study due to different reasons including the changing of the medical center, discontinuing chemotherapy regimen, and death. Consequently, 73 patients were enrolled in this study. The study was approved by the Mazandaran University of Medical Sciences (MAZUMS) Review Board and all patients were given informed consent prior to participation in the study. The demographic and clinical data of patients including age, sex, tumor location, TNM stage (Tumor size, Lymph nodes involvement, Metastasis) and chemotherapy regimen were recorded.

Two milliliters of peripheral blood were collected from each participant and transferred to EDTA-containing tubes and then stored in a freezer at − 80 °C until time of analysis.

Three SNP of *DPYD* gene including IVS14 + 1 G > A, 2194 A > G and 2846 A > T were studied based on PCR-RFLP method.

Genomic DNA was extracted from peripheral blood using the QIA Amp DNA Mini kit (Qiagen, Germany). PCR-RFLP method was used to determine the IVS14 + 1 G > A and 2846 A > T variants applying Mae II and Bse8I restriction enzymes, respectively. Tetra-primer ARMS-PCR was optimized to detect the 2194 G > A variant. Amplification performed with an initial denaturation step (5 min at 95 °C) followed by 35 cycles of 95 °C for 1 min, 60 °C for 1 min for IVS14 + 1 G > A variant, 62 °C for 2846 A > T, 58 °C for 2846 A > T variants, and then 72 °C for 1 min followed by an extension step of 72 °C for 5 min [20].

Side effects experienced by patients including diarrhea, nausea, vomiting, mucositis, peripheral neuropathy, and hair loss were recorded and graded according to CTCAE Version 5.0 [21].

Data analysis was performed using SPSS 24 software. Mean and standard deviation was reported for quantitative data and qualitative dichotomous data were presented as frequency and percent. Comparison of qualitative data was done using Chi-square test. In all cases, *P* <  0.05 was considered a statistically significant difference.

## Results

The demographic and clinical characteristics of patients are presented in Table 1. The colon cancer incidence was more frequent compared to rectal cancer incidence. Most patients had a disease with a TNM stage of 3 or 4 (71%). FOLFOX (72.7%) and FOLFIRI (20.8%) were the most used regimens compared to other chemotherapy regimens (93.5% versus 6.5%).

**Table 1:** Demographic and clinical data of patients (*n* = 88)

Age; year	
Mean	58.7
SD	12.9
Sex; number (%)	
Male	48 (54.5)
Female	40 (45.5)
Site of Cancer; number(%)	
Colon	64 (72.7)
Rectum	24 (27.3)
Tumor stage; number(%)	
Stage 2a	9 (12.3)
Stage 2b	8 (11)
Stage 2c	3 (4.1)
Stage 3a	3 (4.1)
Stage 3b	14 (19.2)
Stage 3c	6 (8.2)
Stage 4a	25 (34.2)
Stage 4b	5 (6.9)
Chemotherapy; number of cycles(%)	
FOLFOX	165 (72.7)
FOLFIRI	47(20.7%)
Capecitabine + Cetuximab	3 (1.3)
Capecitabine	6 (2.7)
FOLOFOX-IRI	5 (2.2)
5-FU	1 (0.4)

*5-FU* 5-Fluorouracil, *FOLFOX: 5-FU* leucovorin and oxaliplatin, *FOLFIRI: 5-FU* leucovorin and irinotecan; colorectal cancer TNM staging was accordant to the American Joint Committee on Cancer staging manual (8th edition)

Additionally, the prevalence of *DPYD* polymorphisms, including IVS 14 + 1 G > A, 2846 A > T, and 2194 G > A, was determined in 73 patients (Table 2).

**Table 2:** The prevalence of *DPYD* gene polymorphisms (*n* = 73)

Polymorphism	Heterozygous	Homozygote	Absence of polymorphism	Total
IVS 14 + 1 G > A	4 (5.5%)	0	69 (94.5%)	73 (100%)
2846 A > T	0	0	73 (100%)	73 (100%)
2194 G > A	0	0	73 (100%)	73 (100%)

IVS14 + 1G > A polymorphism was found in 4 of 73 patients (5.5%), all of which were heterozygous. There were no cases of two other polymorphisms (2846 A > T and 2194 G > A).

The frequencies of chemotherapy-related side effects were summarized in Table 3.

**Table 3:** Adverse drug reactions of chemotherapy regimens

ADR	Chemotherapy regimens								***P***-Value*
		FOLOFOX	FOLFIRI	Capecitabine + Cetuximab	Capecitabine	FOLOFOX + Irinotecan	5-FU	Total	
Diarrhea	Exist	39 (23.6%)	14 (29.8%)	0	1 (16.7%)	2 (40%)	0 (0%)	56 (24.7%)	0.39
	Absent	126 (76.4%)	33 (70.2%)	3 (100%)	5 (83.3%)	3 (60%)	1 (100%)	171 (75.3%)	
	Total	165	47	3	6	5	1	227	
Nausea	Exist	48 (29.1%)	14 (29.8%)	2 (66.7%)	2 (33.3%)	3 (60%)	1 (100%)	70 (30.8%)	0.93
	Absent	117 (70.95%)	33 (70.25%)	1 (33.3%)	4 (66.7%)	2 (40%)	0 (0%)	157 (69.2%)	
	Total	165	47	3	6	5	1	227	
Vomiting	Exist	28 (17%)	9 (19.1%)	1 (33.3%)	2 (33.3%)	3 (60%)	1 (100%)	44 (19.4%)	0.73
	Absent	137 (83%)	38 (80.9%)	2 (66.7%)	4 (66.7%)	2 (40%)	0 (0%)	183 (80.6%)	
	Total	165	47	3	6	5	1	227	
Oral mucositis	Exist	55 (33.3%)	18 (38.3%)	0 (0%)	2 (33.3%)	2 (60%)	1 (100%)	78 (34.4%)	0.53
	Absent	110 (66.7%)	29 (61.7%)	3 (100%)	4 (66.7%)	3 (40%)	0 (0%)	149 (65.6%)	
	Total	165	47	3	6	5	1	227	
Peripheral neuropathy	Exist	159 (96.4%)	36 (76.6%)	3 (100%)	5 (83.3%)	5 (100%)	1 (100%)	209 (92.1%)	< 0.001
	Absent	6 (3.6%)	11 (23.4%)	0 (0%)	1 (16.7%)	0 (0%)	0 (0%)	18 (7.9%)	
	Total	165	47	3	6	5	1	227	
Hair loss	Exist	56 (40%)	24 (58.5%)	2 (66.7%)	5 (83.3%)	3 (75%)	1 (100%)	91 (46.7%)	0.04
	Absent	84 (60%)	17 (41.2%)	1 (33.3%)	1 (16.7%)	1 (25%)	0 (0%)	104 (53.3%)	
	Total	140	41	3	6	4	1	195	

*ADR* adverse drug reaction, *5-FU* 5-Fluorouracil, *FOLFOX* 5-FU / leucovorin and oxaliplatin, *FOLFIRI* 5-FU / leucovorin and irinotecan; *: P-value represents the difference between FOLFOX and FOLFIRI regimens

Patients treated with FOLFOX showed higher rate of peripheral neuropathy (96% versus 76.6%, *P* < 0.001), whereas FOLFIRI regimen was associated with a higher occurrence of hair loss (58.5% vs. 40%, *P* = 0.04). The rate of other adverse effects including diarrhea, nausea, vomiting and oral mucositis were not different. The severity of the side effects experienced with each of the chemotherapy cycles was presented in the Table 4.

**Table 4:** Severity of adverse effects of chemotherapy regimens

ADR	Chemotherapy regimens								***P***-Value*
		FOLOFOX	FOLFIRI	Capecitabine + Cetuximab	Capecitabine	FOLOFOX + Irinotecan	5-FU	Total	
Diarrhea	Grade 1	14 (35.9%)	4 (28.6%)	0 (0%)	1 (100%)	2 (100%)	0 (0%)	21 (37.5%)	0.96
	Grade 2	12 (30.8%)	5 (35.7%)	0 (0%)	0 (0%)	0 (0%)	0 (0%)	17 (30.4%)	
	Grade 3	7 (17.9%)	3 (21.4%)	0 (0%)	0 (0%)	0 (0%)	0 (0%)	10 (17.9%)	
	Grade 4	6 (15.4%)	2 (14.3%)	0 (0%)	0 (0%)	0 (0%)	0 (0%)	8 (14.3%)	
Nausea	Grade 1	33 (68.8%)	7 (50%)	1 (50%)	2 (100%)	2 (66.7%)	0 (0%)	45 (64.3%)	0.32
	Grade 2	14 (29.2%)	7 (50%)	1 (50%)	0 (0%)	1 (33.3%)	1 (100%)	24 (34.3%)	
	Grade 3	1 (2.1%)	0 (0%)	0 (0%)	0 (0%)	0 (0%)	0 (0%)	1 (1.4%)	
Vomiting	Grade 1	17 (6.7%)	5 (55.6%)	1 (100%)	2 (100%)	1 (33.3%)	1 (100%)	27 (61.4%)	0.91
	Grade 2	7 (25%)	3 (33.3%)	0 (0%)	0 (0%)	2 (66.7%)	0 (0%)	12 (27.3%)	
	Grade 3	3 (10.7%)	1 (11.1%)	0 (0%)	0 (0%)	0 (0%)	0 (0%)	4 (9.1%)	
	Grade 4	1 (3.6%)	0 (0%)	0 (0%)	0 (0%)	0 (0%)	0 (0%)	1 (2.3%)	
Oral mucositis	Grade 1	24 (61.4%)	11 (61.1%)	0 (0%)	0 (0%)	1 (50%)	0 (0%)	36 (46.2%)	
	Grade 2	19 (27.3%)	4 (22.2%)	0 (0%)	2 (100%)	1 (50%)	1 (100%)	27 (34.6%)	0.59
	Grade 3	11 (9.1%)	3 (16.7%)	0 (0%)	0 (0%)	0 (0%)	0 (0%)	14 (17.9%)	
	Grade 4	1 (2.3%)	0 (0%)	0 (0%)	0 (0%)	0 (0%)	0 (0%)	1 (1.3%)	
Peripheral neuropathy	Grade 1	89 (56%)	16 (44.4%)	0 (0%)	2 (40%)	4 (80%)	0 (0%)	111 (53.1%)	0.39
	Grade 2	69 (43.4%)	20 (55.6%)	3 (100%)	2 (40%)	1 (20%)	1 (100%)	96 (45.9%)	
	Grade 3	1 (0.6%)	0 (0%)	0 (0%)	1 (20%)	0 (0%)	0 (0%)	2 (1%)	
Hair loss	Grade 1	53 (94.6%)	19 (79.2%)	2 (100%)	5 (100%)	1 (33.3%)	0 (0%)	80 (87.9%)	0.03
	Grade 2	3 (5.4%)	5 (20.8%)	0 (0%)	0 (0%)	2 (66.7%)	1 (100%)	11 (12.1%)	

*ADR* adverse drug reaction, *5-FU* 5-Fluorouracil, *FOLFOX* 5-FU / leucovorin and oxaliplatin, *FOLFIRI* 5-FU / leucovorin and irinotecan; *: *P*-value represents the difference between FOLFOX and FOLFIRI regimens

The grading of most of the adverse reactions was not statistically different for FOLFOX and FOLFIRI regimens, except hair loss. A higher grade toxicity (grade 2) was more common with FOLFIRI compared to FOLFOX (20.8% versus 5.4%, *P* = 0.034). In the case of diarrhea, 5 out of 41 patients who received FOLFIRI (12%) and 13 out of 140 patients who received FOLFOX (9.2%) experienced grade 3/4 diarrhea (*P* = 0.95). Grade 3 nausea was observed only in one case of FOLFOX regimen and there was not any Grade 3 or 4 nausea with FOLFIRI regimen (*P* = 0.32). Vomiting Grade 3/4 was observed in 14.3 and 11.1% of patients received FOLFOX and FOLFIRI, respectively (P = 0.9).

Grade 3/4 oral mucositis occurred in 11.4 and 16.7% of patients treated with FOLFOX and FOLFIRI, respectively (*P* = 0.59). Peripheral neuropathy was a common adverse effect with both FOLFOX and FOLFIRI regimens, but almost all patients experienced Grade 1 or 2 toxicity. Grade 3 toxicity of peripheral neuropathic symptom occurred only in one patient with a FOLFOX regimen (*P* = 0.39).

The incidence of adverse drug reactions considering IVS14 + 1G > A polymorphism was presented in Table 5. Diarrhea, vomiting, oral mucositis, peripheral neuropathy, and hair loss were not different between patients with and without IVS14 + 1G > A polymorphism.

**Table 5:** Frequency of adverse drug reactions in different variants of IVS14 + 1G > A

	Polymorphism IVS14 + 1G > A				
ADR		Heterozygous	Normal	Total	*P*-value
Diarrhea	Exist	1 (1.4%)	16 (21.9%)	17 (23.3%)	0.663
	Absent	3 (4.1%)	53 (72.6%)	56 (76.7%)	
Nausea	Exist	0	30 (41.1%)	30 (41.1%)	0.113
	Absent	4 (5.5%)	39 (53.4%)	43 (58.9%)	
Vomiting	Exist	1 (1.4%)	16 (21.9%)	17 (23.3%)	0.663
	Absent	3 (4.1%)	53 (72.6%)	56 (76.7%)	
Oral mucositis	Exist	2 (2.7%)	27 (37%)	29 (39.7%)	0.522
	Absent	2 (2.7%)	42 (57.6%)	44 (60.3%)	
Peripheral neuropathy	Exist	4 (5.5%)	64 (87.7%)	68 (93.2%)	0.748
	Absent	0	5 (6.8%)	5 (6.8%)	
Hair loss	Exist	4 (5.5%)	37 (50.7%)	41 (56.2%)	0.093
	Absent	0	32 (43.8%)	32 (43.8%)	

All of the patients with IVS14 + 1G > A polymorphism showed hair loss, whereas 50.7% of patients who did not have this polymorphism experienced this side effect (*P* = 0.093).

There was no significant difference between the presence or absence of IVS14 + 1G > A polymorphism with incidence of diarrhea (25% vs. 23.2%; *P* = 0.663), nausea (zero vs. 43.5%; *P* = 0.113), vomiting (25% vs. 23.2%; P = 0.663), oral mucositis (50% vs. 39.1%; *P* = 0.522), and peripheral neuropathy (100% vs. 92.8%, *P* = 0.748).

## Discussion

The aim of the current study was to investigate the prevalence of adverse drug reactions of 5-FU based chemotherapy regimens used in the treatment of CRC and the relationship between three polymorphisms of *DPYD* including IVS 14 + 1G > A, 2846 A > T, and 2194 G > A with the occurrence of adverse drug reactions. Various mutations of the *DPYD* have been reported [22, 23]. In an Italian survey, Mazzuca et al. reported that the prevalence of IVS14 + 1G > A polymorphism in CRC patients was 1.38%, all of which were heterozygous. In comparison with our study, a few percentages of patients had *DPYD* IVS14 + 1G > A polymorphism as compared to the rate of 5.5% in our patients. Similar to our study, all of these polymorphisms were heterozygous. The incidence of severe side effects (Grade 3/4) was similar between the two studies (21.2% versus 18.2% in our study) [24].

Lee et al., Reported an incidence of IVS14 + 1G > A in 2886 Caucasian patients treated with 5-FU containing regimens including FOLFOX and FOLFIRI. Among all patients, 0.94% was heterozygous. Compared to the present study population, a few percent of patients had *DPYD* IVS 14 + 1G > A polymorphism and all of these polymorphisms were heterozygous. In 33.1% of patients (859 cases), severe side effects (Grade 3 and above) were due to 5-FU and 88% of patients with the IVS 14 + 1G > A polymorphism experienced severe side effects compared to patients without this polymorphism (57.1% vs. 18.1%). Common symptoms reported were diarrhea (12%), neutropenia (11.7%), nausea and vomiting (5%), fatigue (4.9%), and mucositis (4.2%) [25]. In this study, the prevalence of diarrhea, nausea, vomiting, oral mucositis, peripheral neuropathy, and hair loss was 24.7, 30.8, 19.4, 34.4, 92.1 and 46.7%, respectively. We did not find any association between the IVS 14 + 1G > A polymorphism and the occurrence of side effects.

Deenen et al. studied the effect of DPYD polymorphisms on the toxicity and effect of Capecitabine on disease progression of 568 Dutch patients with CRC. Patients were received regimes containing Oxaliplatin and Capecitabine and the prevalence of IVS14 + 1G > A polymorphism was 1%, that was less than the amount detected in our study. The results showed a significant correlation between polymorphisms and side effects of Capecitabine. Five out of seven of the patients with IVS141G > A polymorphism experienced diarrhea (71%). All patients with IVS141G > A polymorphism showed Grade 3 or 4 diarrhea symptoms [13].

Unlike our study, He et al. did not see the IVS14 + 1G > A polymorphism in 142 Chinese patients with colorectal and nasopharyngeal cancers [26].

In a study conducted by Uzunkoy et al. in 2007 on 56 patients with CRC cancer in Turkey, they found two cases of IVS14 + 1G > A polymorphism (0.6%), both of which were heterozygous [27]. The rate of IVS14 + 1G > A heterozygous polymorphism was less in their study compared to our population studied.

In a study conducted by Raida et al. in Germany, of 851 Caucasian patients with CRC treated with 5-FU, the prevalence of IVS14 + 1G > A was 0.94%, all of which were heterozygous, and approximately 25% of those who experienced Grade 3 and 4 had this polymorphism [28]. The prevalence of IVS14 + 1G > A polymorphism was higher in our population.

Different populations and races show different prevalence of IVS14 + 1G > A polymorphism in the *DPYD* [29]. In a study on 72 patients in Taiwan, 2.7% of patients had IVS14 + 1G > A polymorphism in the *DPYD* [30]. However, in another study on 262 patients in Taiwan, this polymorphism was not observed [31]. DPD enzyme activity was reported to be higher than normal in North Korea [8] that may influence the efficacy and toxicity of fluoropyrimidine-based adjuvant chemotherapy.

In addition to IVS14 + 1G > A polymorphism, in this study we also examined the presence of other *DPYD* polymorphism including A > T 2846 and 2194 G > A, but these polymorphisms were not observed in our patients. Lee et al. reported that the prevalence of A > T 2846 polymorphism in 2886 Caucasian patients with Grade 3 colon cancer who received FOLFOX and FOLFIRI regimens was about 1.1% [25]. In a study by Terrazzino et al. on 2308 patients, the prevalence of 2846 A > T polymorphisms was 0.2% [32].

Deenen et al. reported that 2194 G > A polymorphism in 568 Dutch patients with CRC receiving the XELOX regimen was about 7%, and they observed the relationship between severity of diarrhea (Grade 3/4) and 2194 G > A polymorphism [13]. But in another study conducted on 142 Chinese patients with colorectal and nasopharyngeal cancers receiving 5-FU-treated regimens, the prevalence of the 2194 G > A polymorphism was reported 1.4% and there was not any relationship with the DPD enzyme activity [26]. Although efforts have been made to determine the association between the *DPYD* and the 5-FU toxicities, it seems that *DPYD* alone can detect approximately 20% of the early side effects of 5FU [15]. Present study shows that there was no significant correlation between IVS14 + 1G > A polymorphism and the profile of side effects of 5-FU in CRC patients. It other words, a major part of the symptoms experienced by patients is independent of *DPYD* polymorphism. But the results of our study should be interpreted cautiously as the sample size of our study was small and the relevant SNPs was only observed in four cases.

## Conclusion

Among the studied polymorphisms, only the IVS14 + 1G > A polymorphism was found in our patients and its prevalence was somewhat higher than the similar studies. Two other polymorphisms including 2194 G > A and 2846 A > T were not found. FOLFOX and FOLFIRI regimens were used more than other regimens. The profile of toxicities of FOLFOX and FOLFIRI regimens was different in some adverse reactions such as peripheral neuropathy and alopecia and we did not observe any relationship between adverse reactions and *DPYD* polymorphism.

## Data Availability

The datasets used and/or analyzed during the current study are available from the corresponding author on reasonable request.

## Abbreviations

5-FU: 5-Fluorouracil
ADR: Adverse drug reaction
CRC: Colorectal cancer
CTCAE: Common terminology criteria for adverse events
DHFU: Dihydrofluorouracil
DPD: Dihydropyrimidine dehydrogenase enzyme
DPYD: DPD gene
FdUMP: 5-fluoro-2′-deoxyuridine 5′-monophosphate
FOLFIRI: 5-FU, leucovorin, and irinotecan
OLFOX: 5-FU, leucovorin and oxaliplatin
PCR-RFLP: Polymerase chain reaction-restriction fragment length polymorphism
SNP: Single nucleotide polymorphisms
TNM disease stage: Tumor size, lymph nodes involvement, metastasis
TS: Thymidylate synthase
XELOX: Capecitabine and oxaliplatin
